# Metagenomic Analyses of the Soybean Root Mycobiome and Microbiome Reveal Signatures of the Healthy and Diseased Plants Affected by Taproot Decline

**DOI:** 10.3390/microorganisms10050856

**Published:** 2022-04-21

**Authors:** Sorina C. Popescu, Maria Tomaso-Peterson, Teresa Wilkerson, Aline Bronzato-Badial, Uyen Wesser, George V. Popescu

**Affiliations:** 1 Department of Biochemistry, Molecular Biology, Entomology, and Plant Pathology, Mississippi State University, Mississippi State, MS 39762, USA; mariat@pss.msstate.edu (M.T.-P.); twilkerson@drec.msstate.edu (T.W.); ar2563@msstate.edu (A.B.-B.); upw2@msstate.edu (U.W.); 2 Delta Research and Extension Center, Mississippi State University, Stoneville, MS 38776, USA; 3 Institute for Genomics, Biocomputing, and Biotechnology, Mississippi State University, Mississippi State, MS 39762, USA; popescu@igbb.msstate.edu

**Keywords:** soybean root, microbiome, mycobiome, taproot decline, *Xylaria necrophora*

## Abstract

Invading pathogens interact with plant-associated microbial communities, which can be altered under the pressure of pathogen infection. Limited information exists on plant–microbe interactions occurring during natural outbreaks in agricultural fields. Taproot decline (TRD) of soybean is an emerging disease caused by *Xylaria necrophora.* TRD disease occurrence and yield loss associated with TRD are outstanding issues in soybean production. We applied nuclear ribosomal DNA Internal Transcribed Spacers and 16S rRNA gene taxonomic marker sequencing to define the composition of the fungal and bacterial communities associated with healthy and diseased soybean roots collected from the Mississippi Delta. The plant compartment was a significant factor regulating taxonomic diversity, followed by the disease status of the plant. TRD impacted the root endophytes, causing imbalances; at the intermediate and advanced stages of TRD, *X. necrophora* decreased mycobiome diversity, whereas it increased microbiome richness. Networks of significant co-occurrence and co-exclusion relationships revealed direct and indirect associations among taxa and identified hubs with potential roles in assembling healthy and TRD-affected soybean biomes. These studies advance the understanding of host–microbe interactions in TRD and the part of biomes in plant health and disease.

## 1. Introduction

Taproot decline (TRD) of soybean is an emerging disease of soybean that has warranted much attention within the past five years. As the name indicates, the taproot is attacked by the pathogen *Xylaria necrophora* [1]. The lower soybean stem and taproot appear black, dry, and brittle. The inner pith is colonized with robust, white mycelium. Diseased plants snap at the soil line, providing a key diagnostic feature in the field. Another diagnostic feature of TRD is the presence of stromata (deadman’s fingers) colonizing soybean or other crop debris remaining from the previous growing season [2]. The foliar symptoms of TRD are similar to those of other root diseases of soybean and include interveinal chlorosis and necrosis. Plant death may occur in the early vegetative stages of soybean development but is not widespread in affected fields. Field symptoms are often localized; symptomatic plants are clustered, one to two meters within a row or adjacent rows. The disease pattern is consistent with a soilborne pathogen, as conidia or ascospore production has not been observed in situ [1]. Since initial observations of TRD, disease occurrence has increased within the United States and is now widespread in soybean production throughout the southern United States [2,3]. Significant yield loss associated with TRD has been reported in Arkansas, Louisiana, and Tennessee [1,4,5]. Early results indicate that host tolerance is the best management approach. To identify management practices that minimize plant destruction and increase yield, we must first understand the pathogen. *X. necrophora* is a novel species, one of only a few *Xylaria* spp. parasitic to plants. The vast majority of *Xylaria* spp. are known as wood decomposers existing as efficient saprobes. Some species survive as nonparasitic endophytes on their hosts, while other *Xylaria* spp. become parasitic on the hosts they colonize. Interestingly, *X. necrophora* is the only species to date that attacks an annual plant such as soybean. Other parasitic *Xylaria* species attack the roots and seeds of perennial plants [6,7]. These parasitic *Xylaria* spp. are considered facultative saprophytes. Biocontrol strategies for fungal diseases emerge as alternative control methodologies [8,9]. However, insufficient knowledge exists on soybean root microbial communities and their inherent potential to suppress TRD [10,11].

Plant roots harbor a diverse microbial community mainly composed of bacteria and fungi. The interactions between the plant host and its microbial communities influence microbiomes’ diversity and taxonomic structure and facilitate essential processes in the host plant, such as nutrient acquisition and resistance to changes in the biotic and abiotic environment [12,13]. The plant microbiota harbors beneficial and pathogenic microorganisms. Microbes colonize the rhizosphere surrounding the plant’s roots and the endosphere, comprising the superficial tissue layers of the root. Studies in experimental model plants and crops have defined microbiomes of various root compartments and soil types [14,15]. Microbiota isolated from the different root compartments shows distinct taxonomic structures and functional composition [16,17], underlining the importance of the complex relationships established among diverse bacterial and fungal communities and their role in shaping the microbiome [18,19,20]. An essential property of microbiomes of many plant species is facilitating plant defense against pathogens and the environmental stress response through mechanisms such as the induction of plant hormones and mobilization and transport of essential nutrients from the soil to the plant. Thus, the microbiota is an expression of the underlying functional relationships of its components with the host plant. Although considered to have a significant impact in determining the outcome of plant–pathogen interactions, the interactions between microbiota and host plants are poorly understood. Knowledge gaps in understanding microorganism–microorganism and host–microorganism interactions are fundamental limitations [21,22].

Recent research has shed light on the dynamics and complex interactions linked to the formation of microbial communities. A need for experimental platforms addressing questions related to understanding the highly dynamic temporal and spatial parameter patterns in the rhizosphere has been recently noted [23,24]. Interestingly, clear spatial and temporal patterns were defined during the assembly of the soybean root microbiome [25]. Indeed, current microbiome niche assembly models postulate the interplay between plant compartments and developmental stages to modulate microbiome community assembly in soybean and grape [26,27]. In humans, research data confirm that the taxonomic composition of disease-associated microbiomes is often distinct from that of healthy individuals [28]. Knowledge of the human microbiome and the factors that influence its composition has been used to understand a particular disease and alter the microbiome deliberately for preventive or therapeutic purposes [29]. Plant pathogens can modify the outcomes of plant–microbiota interactions by promoting enhanced enzyme activity, changing nutrient cycling, regulating the order of microbial succession, inhibiting pathogen growth, and inducing host defense priming [21]. Nevertheless, characterization of the spatial and temporal dynamics of healthy and disease-associated microbiota of plants is still lacking [30,31].

We hypothesized that TRD impacts the compositional range of soybean biomes. Here, roots from healthy and TRD-affected soybean plants at distinct stages of disease (early, moderate, and advanced) were characterized using nuclear ribosomal DNA Internal Transcribed Spacers (ITS) and 16S rRNA taxonomic marker sequencing. We defined the fungal (mycobiome) and bacterial taxa (microbiome) associated with the rhizosphere, endosphere, and agricultural soil collected from the Mississippi Delta. Finally, we inferred microbial co-occurrence networks to gain insights into patterns of association and exclusion among taxa.

## 2. Materials and Methods

***Sample collection.*** Silty loam soil and soybean plants variety AG4632 were collected in July 2019 from a field in the Mississippi Delta, United States (33.430170; −90.868759), at the reproductive (R8) stage of soybean. Soil was collected from two field locations (samples S-A and S-B) and designated as natural soil samples. Four soybean plants were collected, including one healthy plant and three plants showing TRD-specific symptoms; plants were selected randomly, removed from the soil for transport to the lab. TRD symptoms in soybean include chlorotic leaves with mild interveinal chlorosis within the mid-to lower canopy progressing to severe symptoms of foliar chlorosis to necrosis on leaves and stems or total plant blight [32]. Infected soybean plants’ taproot and lateral roots are black, dry, and brittle. Plants were shaken gently to remove loosely adhering particles before being transfered into paper bags. Bulk soil was collected from a depth of 20 cm from the same site. After field collection, all samples were transported to the laboratory and stored at 4 °C before processing. The sampled field was subjected to continuous soybean cultivation under the no-till system and managed according to the Mississippi State Cooperative Extension Service guidelines. 

***Sample Preparation.*** Methods for processing soil and soybean roots were carried out as described [20]. Briefly, soybean shoots were separated from the roots and washed using a phosphate-buffered saline solution supplemented with 200 µL of Silwet (pH 7.0). Soil collected from the outer surface of roots was decanted and transferred to a 50 mL tube. The soil slurry was centrifuged at 3200× *g* for 15 min to precipitate soil particles. The supernatant was removed, and the pellets were resuspended, transferred to 1.5 mL microfuge tubes, and centrifuged at 10,000× *g* for 5 min, after which the supernatant was removed entirely. The resulting pellet was defined as the rhizosphere compartment. Rhizosphere pellets, averaging 250 mg per sample, were flash-frozen in liquid nitrogen and stored at −80 °C until DNA extraction. The bulk soil was processed using the same procedures as the rhizosphere soil. Endophyte samples were obtained from soybean taproots that were cleaned of remaining debris with sterile tweezers and transferred to sterile 50 mL tubes containing sterile phosphate buffer. The roots were sonicated at low frequency for five minutes (five 30 s bursts followed by five 30 s rests), snap-frozen, and stored at −80 °C. Frozen roots were lyophilized in liquid nitrogen before DNA extraction. In total, ten samples including two soil and four root samples were processed to obtain the mycobiome and microbiome datasets for the rhizosphere (R-H, R-S1, R-S2, and R-S3) and endosphere (E-H, E-S1, E-S2, and E-S3).

**DNA extraction and metagenomic sequencing.** Total DNA was extracted using the PowerSoil DNA Kit according to the manufacturer’s instructions (Qiagen, Hilden, Germany) from 250 mg of soil pellets or lyophilized roots. Library preparation followed the Illumina 16S rRNA metagenomic sequencing (https://support.illumina.com/content/dam/illumina-support/documents/documentation/chemistry_documentation/16s/16s-metagenomic-library-prep-guide-15044223-b.pdf, accessed on 17 April 2022) and the ITS Metagenomics Protocols (https://support.illumina.com/content/dam/illumina-support/documents/documentation/chemistry_documentation/metagenomic/fungal-metagenomic-demonstrated-protocol-1000000064940-01.pdf accessed on 17 April 2022) using Nextera XT Index Kit v2. Sequencing was performed at MR DNA (http:/www.mrdnalab.com accessed on 17 April 2022, Shallowater, TX, USA) on an Illumina MiSeq platform following the manufacturer’s guidelines. Briefly, the 16S rRNA gene (V4 variable region–PCR primers 515/806) and the ITS (1–4 regions) were amplified via PCR using the HotStarTaq Plus Master Mix Kit (Qiagen) under the following conditions: 94 °C for 3 min, followed by 28 cycles of 94 °C for 30 s, 53 °C for 40 s, and 72 °C for 1 min, with a final elongation step at 72 °C for 5 min. After amplification, PCR products were verified in 2% agarose gel to determine the success of amplification and their relative abundance. 

**Microbiome meta-analysis, bioinformatics, and statistics.** Sequence data were processed using the Mr. DNA analysis pipeline (http:/www.mrdnalab.com accessed on 17 April 2022, Shallowater, TX, USA). In summary, sequences were joined and depleted of barcodes. Sequences presenting equal or smaller than 150 bp and ambiguous base calls were removed. Sequences were then denoised, and chimeras were removed. Operational taxonomic units (OTUs) were defined by clustering at 3% divergence (97% similarity). Final OTUs were taxonomically classified using BLASTn against a curated database derived from RDPII and NCBI (www.ncbi.nlm.nih.gov, http://rdp.cme.msu.edu, accessed on 17 April 2022). For the analysis of data outputs and statistical analysis, we used the web-based platform MicrobiomeAnalyst module Marker-gene Data Profiling (MDP) (https://www.microbiomeanalyst.ca/, accessed on 17 April 2022) [33]. The following tools in the workflow were used: data filtering and normalization, diversity, and community profiling to obtain alpha/beta diversity and heat trees, comparative, correlation, and enrichment analyses. For data filtering, a low count filter based on prevalence in samples was set at 20%; then, we applied a low variance filter based on the interquartile range (set at 10% limit for the % to remove). For alpha diversity profiling and significance testing, we applied Chao1 and Simpson diversity indices with *t*-test/ANOVA [34]. We used Principal Component Analysis (PCoA) [35], the Bray–Curtis distance metrics, and the PERMANOVA statistical method [36] for beta-diversity profiling. Correlation analyses networks were performed using the SparCC method [37] with the parameters set as follows: permutation at 100, the *p*-value threshold at 0.05, and the correlation threshold at 0.03. A nonparametric univariate statistical comparison using ANOVA/*t*-test with the adjusted *p*-value of 0.05 was used to determine enrichment for selected taxa. Metagenome data were visualized using TreeMap 2019.8.1 [38]. The networks were generated with Cytoscape 3.9.1 [39].

## 3. Results

Replicon sequencing to probe the soybean root microbial and fungal communities from healthy and taproot decline diseased plants

**Figure 1:**
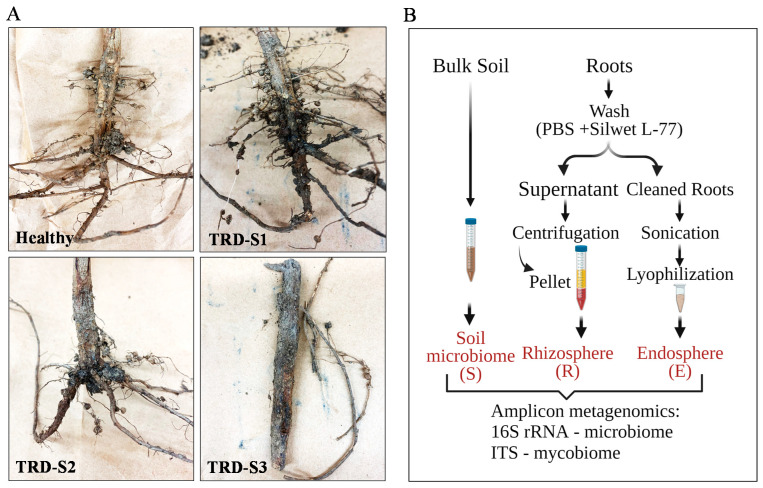
Root material and analysis scheme to investigate the microbiome and mycobiome of healthy and diseased soybean roots. (**A**) Soybean roots were collected from healthy plants and plants showing symptoms of taproot decline (TRD) at incipient (S1), moderate (S2), and advanced (S3) stages of taproot decline disease. Representative images of the roots before processing are shown. (**B**) Root samples and bulk soil collected from the same field site were processed to obtain the microbial and fungal communities of the soil (S), root rhizosphere (R), and endosphere (E). The composition of all samples was investigated using amplicon metagenomics (16S rRNA sequencing and internal transcribed spacer (ITS) regions 1–4 sequencing).

Healthy and TRD symptomatic soybean plants grown in a field with a high incidence of TRD and bulk soil from a depth of 20 cm from the same site were collected for microbiome and mycobiome analysis. The sampled field was subjected to continuous soybean cultivation under the no-till system and managed according to the Mississippi State Cooperative Extension Service guidelines. All plants collected were at the reproductive (R8) stage when TRD symptoms worsened [1]. The TRD symptomatic plants included samples at incipient (S1), moderate (S2), and advanced (S3) stages of infection with *X. necrophora*. TRD severity was assessed according to [2]. S1 samples displayed the characteristic mild leaf interveinal chlorosis within the lower canopy, whereas in S2 samples, the chlorosis advanced to mid- and upper canopy leaves and stems; S3 samples displayed total plant blight. An examination of the S1–S3 alongside healthy roots revealed characteristic TRD symptoms in S1, S2, and S3 samples with symptoms that increased in severity from S1 to S3 (i.e., shortening of the taproot, reduced number of lateral roots, root dryness and brittleness, and areas of a black appearance characteristic to abundant *X. necrophora* growth) (Figure 1A). 

The taxonomic diversity of soybean roots and bulk soil fungal and microbial communities was investigated by sequencing (Illumina) amplicons derived from ITS and 16S ribosomal RNA (rRNA) amplification. For the analysis, two bulk soil samples were processed to obtain the soil communities; soybean roots were processed to separate four rhizosphere and four endosphere fractions from separate roots (Figure 1B). The ITS amplicon sequencing of the ten samples yielded 1,174,764 high-quality total reads, with a median of 104,845 sequences per sample (Supplementary Figure S1A and Supplementary Table S1). Overall, OTUs were categorized into four kingdoms: 53% Fungi, 45% Eukaryota, 1.34% Metazoa, and 0.29% Viridiplantae (Supplementary Figure S1B). The dataset was normalized to an even sequencing depth of approximately 44,000 sequences (Supplementary Figure S1C) prior to statistical and taxonomic analyses. The 16S rRNA sequencing produced, on average, 25,560 reads per sample (Supplementary Figure S1D and Supplementary Table S1), out of which a majority (93%) represented bacteria (Supplementary Figure S1E). We used the microbial meta-analysis pipeline [33] of the abundance (count) tables computed for fungi and bacteria kingdoms to analyze the soybean root mycobiome and microbiome in-depth.

The biological niche is a significant factor modulating the diversity of mycobiome and microbiome communities. 

**Figure 2:**
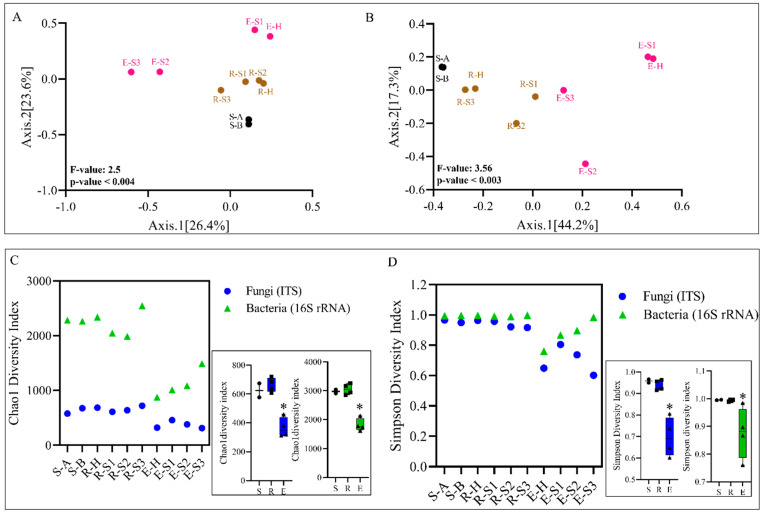
Alpha and beta diversity of the mycobiome and microbiome of soybean roots. (**A**,**B**) Constrained Analysis of Principal Coordinates (PCoA) of ITS (**A**) and 16S (**B**) diversity in the soil (S-A and S-B), rhizosphere (R-H, R-S1 to R-S3), and endosphere (E-H, and E-S1 to E-S3) of plants healthy or at diverse stages of taproot decline disease. Insets show PCoA 3D plots. Cumulative-sum scaling transformed reads were used to calculate Bray–Curtis distances, and significance was assessed by PERMANOVA (*p*-value < 0.05). (**C**,**D**) Alpha diversity was calculated for 16S rRNA and ITS datasets using the Chao1 and Simpson diversity measures. Insets show cumulative values for soil, rhizosphere, and endosphere samples. The significance of the alpha diversity profiling was assessed through ANOVA (*p*-value < 0.05). Asterisks (*) show statistical significance.

We first used unconstrained principal coordinate analysis (PCoA) to profile sample diversity and quantify the major components driving differences in the composition of the fungal and bacterial communities among samples (beta-diversity). PCoA resulted in three distinct clusters—bulk soil, rhizosphere, and endosphere—for the ITS (Fungi) and 16S (Bacteria) datasets. In the mycobiome PCoA, axis 1 (26% of the overall variation) also separated heavily symptomatic E-S2 and E-S3 from the rest of the samples, suggesting that TRD affects the endophyte fungal community composition mainly. Rhizosphere samples showed high similarity among themselves, irrespective of the TRD symptomatic status of the originating root sample, suggesting a lesser effect of TRD on the rhizosphere than on the endosphere (Figure 2A). PCoA axis 2 (24% of the overall variation) mostly separated the bulk soil from the endosphere and rhizosphere samples (Figure 2A). In the case of bacteria, and to a more considerable extent than for fungi, a well-defined separation of the soil, rhizosphere, and endosphere occurred on axis 1 (explaining 44% of the overall variation) (Figure 2B), suggesting that the biological niche is a stronger determinant of variation among bacterial communities than the TRD status of the sample. These observations were largely recapitulated by the hierarchical clustering of pairwise Bray–Curtis dissimilarities among all samples. In this analysis, the four rhizosphere samples clustered together and apart from the four endosphere samples for both the mycobiome and microbiome; in addition, the endosphere H and S1 stage samples clustered apart from the S2 and S3 samples for the mycobiome (Supplementary Figure S2A) and microbiome (Supplementary Figure S2B). 

Analysis of two alpha diversity indices, CHAO1 and Simpson, revealed significant differences across samples from diverse niches, with the endosphere showing an overall lower fungal and bacterial OTU richness than the soil and rhizosphere; moreover, in the case of bacteria, both diversity indices showed an increasing diversity trend among endosphere samples that correlated with TRD status (Figure 2C,D). 

TRD severity is reflected in the taxonomic composition of root endophytes. 

**Figure 3:**
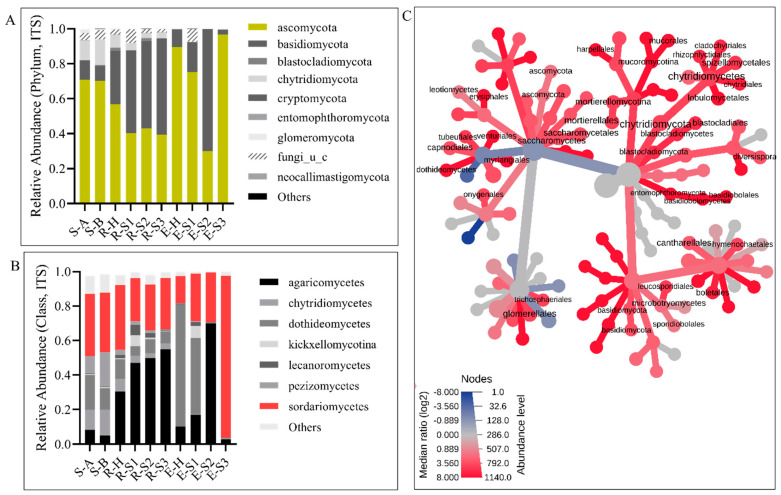
The effect of taproot decline disease on the soybean root mycobiome. (**A**,**B**) The bar graphs report the relative abundance of fungal taxa at the phylum (**A**) and class level (**B**) in bulk soil (S-A and S-B), rhizosphere (R-H, R-S1 to R-S3), and endosphere (E-H and E-S1 to E-S3) mycobiomes of healthy and TRD-symptomatic soybean. Only taxa with the highest abundance are shown. (**C**) Heat tree visualization of taxonomic differences between rhizosphere and endosphere mycobiomes. Blue and red indicate that corresponding taxa are lower and higher in the rhizosphere compared with the endosphere. The color gradient and the size of the node, edge, and label are based on the log_2_ ratio of median abundance.

We analyzed the taxonomic profiles of the root samples. We found that measurable fungi OTUs were distributed across nine phyla (Figure 3A and Supplementary Table S2). Ascomycota was the most abundant phylum in the soil (70%), followed by Chytridiomycota (13%). Ascomycota was distributed among Sordariomycetes (i.e., Hypocreales 11%, Glomerellales 11%, and Xylariales 6%), Pezizomycetes (20%), and Dothideomycetes (11%) (Figure 3B). *Fusarium* sp. (Hypocreales), *Gibellulopsis nigrescens* (Glomerellales), *Microdochium* sp. (Xylariales), and *Ascobolus crenulatus* were among the soil dominant taxa (Supplemental Figure S3A). Almost equal distributions of Ascomycota and Basidiomycota were found in the rhizosphere (44 and 48%, respectively), which, similar to the bulk soil, showed remarkable similarities in their taxonomic profiles (Figure 3A). The Agaricomycetes (Cantharellales 31% and Agaricales 58%) were the most abundant Basidiomycetes; Sordariomycetes (i.e., Hypocreales 25% and Xylariales 13%) were the dominant Ascomycetes (Figure 3B). *Ceratobasidium* sp., *Coprinopsis spilospora*, and *Mycena maurella* dominated the Basidiomycota, whereas *Fusarium* sp. dominated the rhizosphere mycobiota (Supplementary Figure S3B).

**Figure 4:**
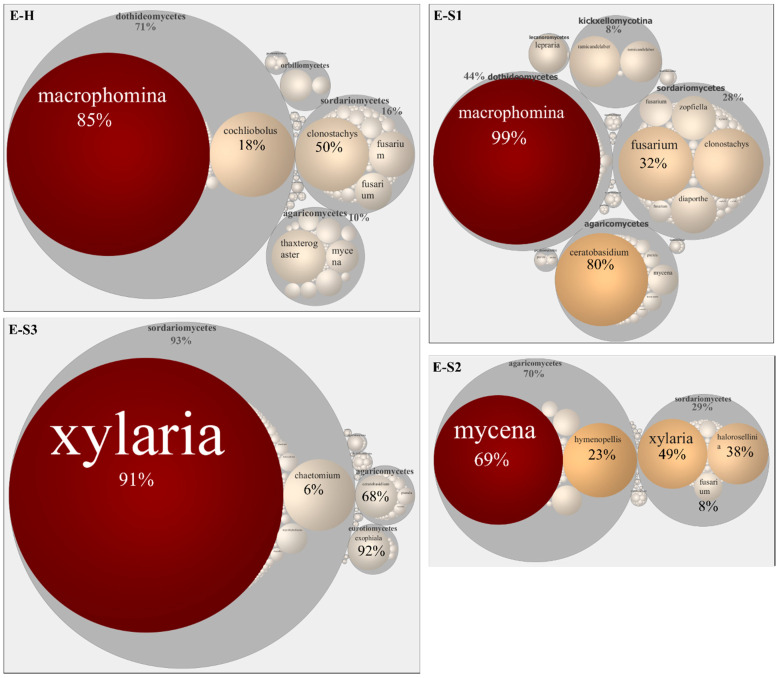
Taxonomic circular maps of soybean fungal root endophytes. Taxonomic analyses of the endophytes (E) in healthy (H) and TRD-affected roots (S1 to S3) are based on ITS rRNA sequencing and OTUs with the highest abundance (*n* > 0.2%). The taxa are grouped by class; the labels show the most abundant genera. The size of the map circles is proportional to the reads number. The arrows point to the position of Xylariaceae. Numbers depict percentages of selected taxonomical categories.

An OTU enrichment analysis to identify core mycobiomes paralleled our taxonomic study and identified Ascomycota, Basidiomycota, and Chytrydiomycota as major core groups. We confirmed genera enrichment, including *Ascobolus*, *Phoma*, and *Fusarium* in bulk soil (Supplementary Figure S4A) and *Ceratobasidium*, *Fusarium*, and *Mycena* in the rhizosphere (Supplementary Figure S4B). A heat tree analysis captured differential abundance patterns between endosphere and rhizosphere mycobiomes; OTUs classified as Glomerellales, Chytridiomycota, Cladochytriales, and Saccharomycetales, among others, were enriched in the rhizosphere (Figure 3C). The endosphere samples showed higher variability than the rhizosphere, reinforcing our observation that TRD affects the endosphere mycobiome to the most considerable extent (Figure 4). Botryospheriales (*Macrophomina phaseolina*) dominated E-H and E-S1 (58% and 67%, respectively). Most E-S2 OTUs were classified as Basidiomycota (70%), among which Agaricomycetes such as *Micena maurella* predominated, followed by Ascomycota (30%), among which Xylariaceae represented 25% of the OTUs. E-S3 was composed almost exclusively (93%) of Sordariomycetes, with Xylariales dominating this class (91%). Xylariaceae had a minor representation in E-H and E-S1 (below 1%), although the *Xylaria* sp. abundance was slightly higher in E-S1 than in E-H (83% versus 69% of all Xylariaceae, respectively). When considering the Xylariaceae solely, E-S2 was dominated by *Xylaria* sp. (57%) and *Harolosellinia* sp. (43%), whereas *Xyaria* sp. was predominant (99.6%) in E-S3. 

**Figure 5:**
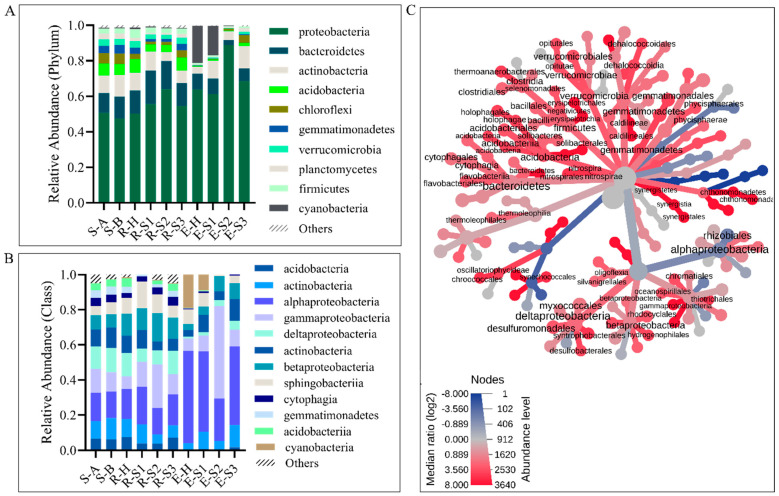
The effect of taproot decline disease on the soybean root microbiome. (**A**,**B**) The bar graphs report the relative abundance of bacterial taxa at the phylum (**A**) and class level (**B**) in bulk soil (S-A and S-B), rhizosphere (R-H, R-S1 to R-S3), and endosphere (E-H and E-S1 to E-S3) microbiomes of healthy and TRD-symptomatic soybean. Only taxa with the highest abundance are shown. (**C**) Heat tree visualization of taxonomic differences between rhizosphere and endosphere microbiomes. Blue and red indicate that corresponding taxa are lower and higher, respectively, in the rhizosphere as compared with the endosphere. The color gradient and the size of the node, edge, and label are based on the log_2_ ratio of median abundance.

**Figure 6:**
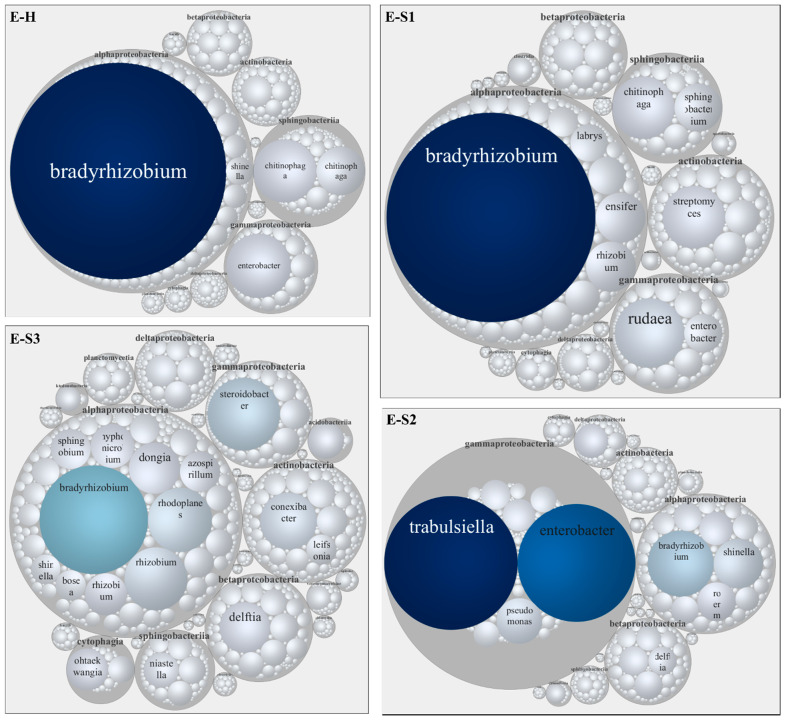
Taxonomic circular maps of soybean bacterial root endophytes. Taxonomic analyses of the endophytes (E) in healthy (H) and TRD-affected roots (S1 to S3) are based on 16S rRNA sequencing and OTUs with the highest abundance (*n* > 0.2%). The taxa are grouped by class; the labels show the most abundant genera. The size of the map circles is proportional to the reads number. Numbers depict percentages of selected taxonomical categories.

The taxonomic analysis of the 16S rRNA data reinforced the similarity of the bulk soil and rhizosphere on the one hand and the variability among endosphere samples on the other (Supplementary Table S2). Proteobacteria (represented by Alphaproteobacteria, Deltaproteobacteria, Gammaproteobacteria, and Actinobacteria) and Bacteroidetes (*Cytophagia* and *Sphingobacteriia*) dominated the bacterial communities of bulk soil and root rhizosphere (Figure 5A,B). *Steroidobacter* sp. (7% of Proteobacteria), *Gemmatimonas* sp. (4% of Gemmatimonadetes), and *Acidobacterium* sp. (4% of Acidobacteria) were the most abundant soil taxa (Supplementary Figure S3C). *Acidobacterium* sp. (4% of Acidobacteria), *Pelobacter* spp. (3% of Proteobacteria), and *Chitinophaga* sp. (3% of Bacterioidetes) were the most abundant rhizosphere taxa (Supplementary Figure S3D). These taxa were also identified as components of the core microbiomes of the soil and rhizosphere (Supplementary Figure S4), respectively. Differential abundance patterns between endosphere and rhizosphere microbiomes were detected and visualized using heat trees (Figure 5C). Bacterioidetes, *Acidobacteria*, Firmicutes, and Gematimonadales were among the taxa enriched in the rhizosphere relative to the endosphere. At moderate and advanced TRD, Gammaproteobacteria dominated the E-S2 endosphere (42%), with Entereobacteriales such as *Trabulsiella* and *Enterobacter* the most abundant genera; Alphaproteobacteria had the highest relative abundance in E-S3 (Figure 5C). On the other hand, the nitrogen-fixing symbiotic bacterium *Bradyrhizobium* had the highest abundance in the endosphere microbiome; *Bradyrhizobium*, the most abundant genus of Rhizobiales in E-H (90%) and E-S1 (77%), was followed by Cyanobacteria, with *Halospirulina* as the dominant taxon (>99% in both E-H and E-S1) (Figure 6 and Supplementary Figure S4). Notably, in E-S2 and E-S3, *Bradyrhizobium* decreased markedly in abundance (35% and 36% of Rhizobiales, respectively); *Shinella* spp. and *Agrobacterium tumefaciens* increased in the relative abundance in E-S2, whereas *Rhodoplanes* sp. and *Rhizobium* sp. were the abundant Rhizobiales in E-S3 (Supplementary Figure S4). 

Describing taproot decline disease through imbalances in the fungal and microbial communities:

We observed imbalances in the diversity and taxa abundance of fungal and microbial communities, more apparent at advanced TRD stages. These observations prompted us to search for possible statistically significant changes in fungal and microbial communities between the ‘Healthy’ (E-H and R-H) and ‘TRD’ (E-S1 to S3 and R-S1 to S3) cohorts. We inferred a correlation network using SparCC (*p*-value threshold 0.05 and correlation threshold 0.3) to identify organisms that reached statistically significant associations with the disease phenotype in the soybean mycobiome and microbiome (Supplementary Figure S5A,B). Slightly more of the identified interactions supported significant co-exclusion between taxa (negative correlation, 392 interactions) than co-occurrence (positive correlation, 386 interactions) (Supplementary Table S2). 

**Figure 7:**
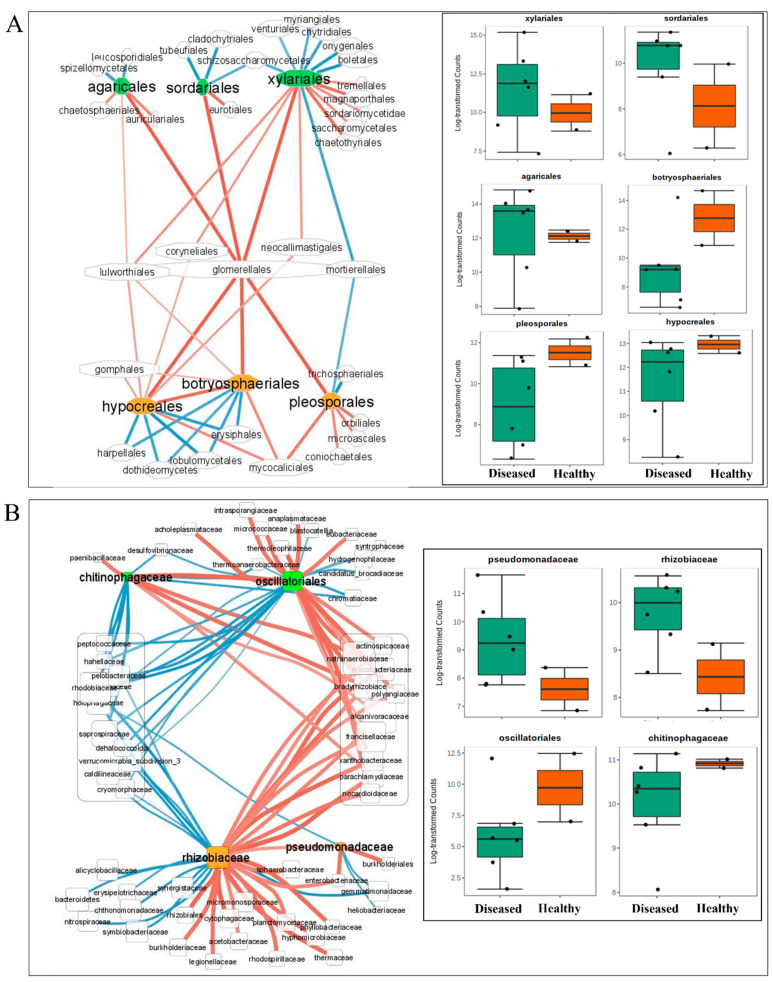
Inference of taxa associations in the healthy and TRD soybean root biomes. (**A**,**B**) Correlation networks based on Pearson correlation of ITS (**A**) and 16S (**B**) rRNA reads extracted from metagenomes of healthy and TRD symptomatic plants with nodes representing taxa and edges representing correlations between taxa pairs. A connection between nodes stands for a statistically significant (*p* < 0.05) correlation. Node size is proportional to reads abundance. Blue edges show a negative correlation, whereas red shows positive correlations. Box plots of taxa showing a differential abundance in TRD versus healthy controls to the right.

We selected taxa with the highest abundance and lowest *p*-values values to generate microbial interaction networks. In these networks, each node represents a fungal or bacterial clade (taxon or group of taxa) connected by edges that were weighted by the significance (*p*-values) of their association. Figure 7A (for fungi) and B (for bacteria) provide the final networks and the associated data. Xylariales, Sordariales, and Agaricales were among the most highly connected taxa co-occurring in the ‘TRD’ condition and showed a low abundance in the ‘Healthy’ cohort. Botryosphaeriales, Pleosporales, and Hypocreales, co-occurring in the ‘Healthy’ condition, became depleted in the ‘TRD’ cohort (Figure 7A and inset). These taxa showed positive or negative relationships with other highly connected taxa; both Xylariales and the ‘Healthy’-enriched Hypocreales were positively correlated with Glomerellales, whereas both Xylariales and the ‘Healthy’-enriched Pleosporales were negatively correlated with Mortierellales. The analysis retrieved positive and negative taxa correlations among bacterial taxa as well (Supplemental Figure S5B and Supplementary Table S2). Pseudomonadaceae and Rhizobiaceae increased significantly in ‘TRD’ compared to ‘Healthy,’ whereas Chitinophagaceae and Oscillatoriales were more abundant in ‘Healthy’ than in the ‘TRD’ cohort (Figure 7B and inset). Similar to our observations for the fungal communities, these nodes were connected by a few direct relationships. 

## 4. Discussion

The plant roots constitute a complex habitat harboring fungal and microbial communities that co-exist in diverse niches and plant compartments. The stability of mixed communities is detemined by the antagonistic and mutualistic interspecies interactions occurring within individual biomes and over an evolutionary timescale [40]. However, perturbations such as pathogen invasion can disrupt the equilibrium of the system and favor species with pathogenic potential that drive the development of disease [41]. While the effect of pathogens in human- and animal-associated biomes has been extensively explored [42,43], we only have limited information on the processes underlying the transition from a healthy plant-associated biome to a pathobiome [41,44,45,46]. Moreover, only scarce information exists on the diverse types of plant–microbe interactions occurring during natural outbreaks of known pathogens in agricultural fields [47]. 

This study assessed the root mycobiome and microbiome of soybean plants grown in a farming field site in the Mississippi Delta. We aimed to evaluate the mycobiota and microbiota taxonomic structures and richness while testing the impact of *X. necrophora* infection on the temporal dynamics of the fungal and bacterial communities (Figure 1). Our results provide evidence that: (1) the topology of fungal and bacterial microbiota (rhizosphere and endosphere) is the primary determining factor in community assembly, (2) TRD has a strong effect on the taxa richness of the endosphere mycobiome and negligible effects on the rhizosphere, and (3) at advanced stages, TRD leads to a re-organization of the root mycobiome and microbiome that favors specific microbial associations. 

Evidence of the crucial role of spatial information in microbial niche differentiation is ample [25,48,49]. Our results reinforce previous observations and bring new information on the impact of TRD on niche differentiation. We found that the plant compartment/niche is a potent discriminatory factor for the assembly of both fungal and bacterial microbiomes of soybean roots, irrespective of the healthy/diseased status of the plant (Figure 2A,B). This observation was supported by the diversity index analysis (Figure 2C,D), showing that endophyte communities’ richness is lower than that of the rhizosphere. The rhizosphere provides an abundance of plant-derived carbohydrates and exudates that stimulate microbial biomass growth [48,50]. Endophytes, recruited from the rhizosphere microbiome or accessing plant tissues via lesions, were previously shown to form a microbiota distinct from the rhizosphere and bulk soil with a comparatively lower level of taxa diversity [18,51]. We found that the rhizosphere mycobiota was enriched in Glomerellales (Sordariomycetes), Chytridiomycetes, and Saccharomycetales. Most of these taxa were identified in other rhizospheric microbiomes as well [52]. Bacterioidetes, *Acidobacteria*, Firmicutes, and Gematimonadales were among the bacterial taxa prevalent in the rhizosphere, supporting previous observations [53,54,55].

Interestingly, the fungal and microbial communities responded distinctly to severe TRD. The PCoA analysis separated heavily symptomatic (E-S2 and E-S3) from the mildly symptomatic and healthy samples, suggesting that a high pathogen load displaces the ‘healthy’ endosphere taxa (Figure 2A). In these samples, TRD dramatically decreased the richness of the fungal endophyte community. Agaricomycetes (70%) and Sordariomycetes (29%, out of which 49% was *Xylaria* sp.) comprised the bulk of fungi in E-S2; at 91% of all Sordariomycetes, *Xylaria* sp. almost exclusively represented the E-S3 endophytes (Figure 4). On the contrary, we observed an increase in the diversity of bacterial endophytes that correlated with the increasing TRD severity; TRD increased the number of detected bacteria classes from 11 (in healthy samples) to 20, 18, and 26 in E-S1, E-S2, and E-S3, respectively (Figure 6). The observed increase in microbial diversity under the severe TRD condition could be driven by the biotic stress pressure on microbial community assemblage as a way to increase plant fitness [56] or, more likely, by dysbiosis, defined as an imbalance of the microbiome causing an abnormal increase in selected minor taxa and decrease in the dominant core species [57]. Indeed, we observed adverse effects of TRD on the high abundance core genus *Bradyrhizobium* (Figure 6). Of note, taxonomic differences between E-S2 and E-S3 suggest the displacement of the abundant fungal and bacterial taxa by the pathogen and the establishment of opportunistic taxa. A similar situation was observed in soils infested with the pathogenic soybean cyst nematode; the parasitic nematodes increased the diversity of the endophytic fungal communities in soybean roots [58]. Moreover, diversity shifts driven by infection with the bacterial pathogen *Xylella fastidiosa* were also observed in bacterial and fungal communities associated with the grapevine xylem. Higher microbial diversity was documented in vines with moderate disease symptoms compared to the severely symptomatic vines [59].

Aside from *Xylaria,* several other fungal genera responded positively to advanced TRD stages. From the Basidiomycota, the Agaricales *Mycena maurella* and *Hymenopellis* dominated E-S2 [60] (48% and 15%, respectively). *M. maurella* belongs to the widespread saprotrophic genus *Mycena*; although not expected to be found as a root endophyte, *Mycena* associates with multiple plant hosts and was suggested to be an opportunistic root pathogen [61]. Interestingly, *Hymenopellis,* growing mainly on dead or buried hardwoods, was found to be a rich resource of bioactive compounds with antimicrobial, antioxidative, anti-inflammatory activities [62]. Among the Sordariomycetes (Ascomycota), the understudied endophyte *Halorosellinia* classified in Xylariaceae was second in abundance after *Xylaria* sp. Another endophyte, *Chetomium erectum*, represented 6% of the Ascomycota in E–S3. Species of *Chaetomium* are widely distributed in nature and generally characterized as high producers of enzymes that catalyze the degradation of cellulose, lignin, and other plant-derived organic compounds [63]. The substrate and host affinity of these high-abundance taxa identified in advanced TRD samples are diverse; however, they suggest enrichment in fungal species able to act as aggressive, opportunistic invaders.

Soybean develops symbiotic associations with diverse nitrogen-fixing rhizobia, including *Bradyrhizobium* and *Rhizobium* species [64]. Rhizobiales, although present in both the rhizosphere and endosphere, were also significantly enriched in the soybean endosphere microbiome (Figure 5C), paralleling previous observations [60]. The positive effect on bacterial communities’ richness in intermediate and advanced TRD correlated with a significant decrease in the abundance of the dominant core taxa *Bradyrhizobium* spp. (Alphaproteobacteria) in E-H and E-S1 (42 and 30% reduction, respectively); on the other hand, advanced TRD correlated with an increased abundance of *Rhodoplanes* and *Steroidobacter* (Figure 6). *Rhodoplanes* sp., classified as a possible N-fixing bacteria, is a top colonizer of the rhizosphere soil associated with oilseed rape [65,66]. *Steroidobacter* sp. are characterized as bacteria with a high capacity to degrade organic compounds and nitrification/denitrification properties [67,68]. Both genera were previously found associated with microbiomes [65,69]. 

Fungal and bacterial association networks were inferred from the observational data (Figure 7). We characterized microbial co-occurrence and co-exclusion patterns throughout the healthy and diseased soybean roots. The analysis of these microbial networks provides initial observations into community organization and putative functional interactions among taxa. Several hub taxa were identified, characterized by a large number of connections and dominance within the ‘healthy’ or ‘diseased’ states. Notably, some of the hubs acted as connectors between multiple healthy- and TRD-associated microbes. For example, no direct connections were observed between the diseased and healthy sub-networks for the fungal and bacterial taxa. However, the order Glomerellales served as the main ‘connector’ of fungal taxa (Figure 7A), whereas multiple taxa mediated bacterial associations (Figure 7B). We hypothesize that hub microbes may act as critical determinants in the transition from healthy to diseased root communities. 

Positive correlation relationships may describe mutualistic, synergistic associations, among other types, including nutritional strategies (e.g., saprophyte or parasite) and dependencies among taxa (e.g., cross-feeding). Negative correlations, likewise, may signal competitive relationships due to the production of toxins or predator–prey relationships. In the fungal association network, the top-scoring orders included Xylariales, Sordariales, and Agaricales; these fungi were most abundant in communities associated with the TRD-infected soybean roots. On the other hand, Botryospheriales, Hypocreales, and Pleosporales were found to be associated with healthy roots. Glomerellales showed positive correlations with all the above groups, whereas Mortierellales were negatively correlated with Xylariales and Pleosporales. Although the current study is associative and does not describe the type of interaction for these microbial associations, several of the observed associations are indirectly supported by published research. For instance, Xylariales, Pleosporales, Hypocreales, and Glomererellales—taxa with positive correlation in the network—were found most abundant and diverse in a large set of fungal isolates [70], indicating an ability to co-exist in mixed cultures. Pleosporales were isolated from various habitats, growing as saprophytes, endophytes, or parasites on fungi or insects [71]. Most species of *Glomerella* are necrotrophic, feeding on dead plant tissue [70]; similarly, Sordariales dominate in fungal communities associated with residue decomposition in arable soils [72]. Mortierellales showed negative correlation values with both Pleosporales and Xylariales; although Mortierelalles includes saprophytic fungi, some species produce antifungal and antibacterial secondary metabolites [73] that may exert antagonistic effects on competitors. Xylariales and Agaricales showed a positive correlation with pest suppressiveness in studies of nematode pests [74,75], suggesting causative associations between these fungal orders. Chitinophagaceae and Oscillatoriales appeared as top-scoring taxa in diseased roots in the bacterial association networks showing a strong positive correlation. Chitinophagaceae are known to provide a rich carbon and nitrogen source for soil microorganisms [76]. Interestingly, metagenomics followed by network inference found that Chitinophagaceae were enriched in the beetroot endosphere post-infection and in a synthetic microbial community that suppressed root disease [77], attesting to their capacity to survive changes in the structure of root biota. Likewise, in healthy roots, Rhizobiaceae and Pseudomonadaceae showed a strong positive correlation, suggesting a capacity for co-existence in the soybean rhizobiome.

## 5. Conclusions

This study investigated the composition of mycobiota and microbiota in soybean roots from plants at the reproductive stage grown in the Mississippi Delta. Soybean plants, healthy or showing TRD symptoms from infection with *X. necrophora*, were selected for comparative analysis. The analysis revealed that, surprisingly, rather than the disease status, the natural niche was the main factor driving differences in the diversity of the fungal and bacterial communities among samples, indicating remarkable stability of natural communities under pathogen pressure. However, the endosphere’s fungal and bacterial communities differed in their composition in samples with high *X. necrophora* load. Advanced disease correlated with increased abundance and diversity of bacteria, whereas it had the opposite effect on fungal diversity, suggestive of *X. necrophora*-induced changes in the microbiota dynamics. Core genera in the rhizosphere mycobiome included *Ceratobasidium*, *Fusarium*, *Coprinopsis*, and *Mycena*. *Xylaria* sp. dominated in the endosphere of heavily symptomatic plants. Likewise, *Chitinophaga*, *Pelobacter*, *Acidobacterium*, and *Pseudomonas* were representative genera of the rhizosphere microbiome. In the endosphere, *Bradyrhyzobium* sp. was the most abundant genus in healthy and diseased roots, albeit its relative abundance steeply decreased in advanced TRD. 

We generated a catalog of co-occurrences and co-exclusions for the mycobiome and microbiome of the soybean roots, represented as networks with positive and negative correlations among taxa. These networks provide initial information on the taxonomic structure of biomes under the pathogen pressure and identify fungal and bacterial taxa with high potential in determining the stability of communities. We hypothesize that the soybean root’s shift from healthy to diseased states is accompanied by complex interactions among diverse fungal and bacterial taxa, whereby species with pathogenic potential overgrow. Nevertheless, the predictions advanced by our work require future testing in natural and synthetic biomes. Understanding the principles underlying complex multi-species assemblages and the factors determining their stability and dynamics lays the foundation of sustainable agriculture [78,79].

## Data Availability

The datasets generated during the study are publicly archived. The Illumina MiSeq sequence raw reads are available in the NCBI Sequence Read Archive (SRA) under BioProject PRJNA821528.

## Supplementary Materials

The following supporting information can be downloaded at: https://www.mdpi.com/article/10.3390/microorganisms10050856/s1, Figure S1: (A) Library size overview for the ITS (A) and 16S rRNA (D) sequencing. For data filtering, features with low counts and prevalence in samples (<10%) were removed. (B) Pie charts showing percentages of kingdoms identified by ITS (B) and 16S rRNA (E) sequencing. (C) Rarefaction curves (minimum library size, total sum normalization). The relationship between the number of OTUs observed and the sequencing depth is shown for all samples; Figure S2: Dendrogram analysis of biome data for mycobiome (A) and microbiome (B) using Bray-Curtis Index as a distance measure and Ward clustering algorithm.; Figure S3: (A–D) Circular maps reporting taxonomic classification of fungal and bacterial taxa in bulk soil (A,C) and rhizosphere (C,D). Taxonomic differences are based on ITS and rRNA sequencing and OTUs with the highest abundance (*n* > 0.2 %) for fungal taxa (A,B) and bacterial taxa (C,D). The taxa are grouped by phylum; the labels show class, order, and species. The size of the map circle is proportional to the reads number; Figure S4: Core biomes. Taxa detected containing classes with the highest prevalence (>20%); count data were transformed to relative abundance for the analysis of the core mycobiome (A) in bulk soil, rhizosphere, and endosphere and the respective core bacterial microbiomes (B); Figure S5: Average reads numbers are based on 16S rRNA sequencing for the E-H (healthy) and TRD-symptomatic samples (E-S1, E-S2, and E-S3). Labels show family and species. Numbers depict percentages of selected taxonomical categories. The size of the map circle is proportional to the reads number; Figure S6: A correlation network was generated using the SparCC algorithm, with nodes representing taxa at the genus level and edges representing correlations between taxa pairs. Co-occurrence networks are based on the Pearson correlation of ITS (A) and 16S (B) rRNA reads extracted from metagenomes of healthy (purple) and TRD symptomatic (orange) plants and soil (green). A connection between nodes stands for a statistically significant (*p* < 0.05) correlation with magnitude (correlation threshold) r > 0.5. Network parameters: *p*-value threshold 0.05, correlation threshold 0.3; Table S1: ITS counts cumulative data and 16S rRNA counts cumulative data; Table S2: Mycobiome taxonomy analysis and Microbiome taxonomy analysis; Table S3: Fungal correlation network data and Bacterial correlation network data.
