# Supplementary material for: Metagenomic Analyses of the Soybean Root Mycobiome and Microbiome Reveal Signatures of the Healthy and Diseased Plants Affected by Taproot Decline
Source: Microorganisms. 2022 Apr 21;10(5):856. doi: 10.3390/microorganisms10050856 (PMC9143508; doi:10.3390/microorganisms10050856)
Supplement: Supplementary file 1 [file microorganisms-10-00856-s001.zip › Supporting figures.pdf]

Supporting figures

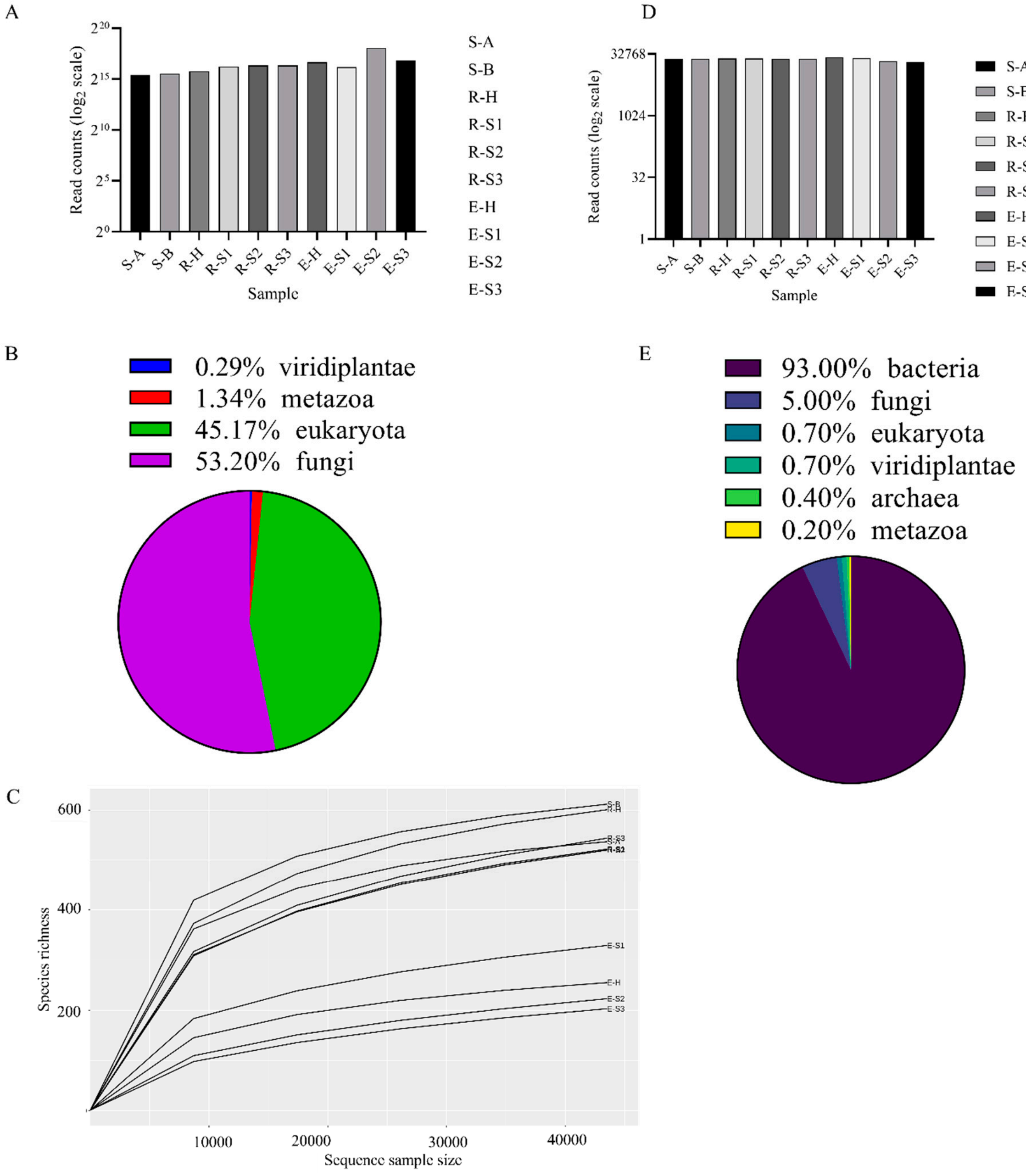

Supplemental Figure S1. A) Library size overview for the ITS (A) and 16S rRNA (D) sequencing. For data filtering, features with low counts and prevalence in samples (< 10%) were removed. B) Pie charts showing percentages of kingdoms identified by ITS (B) and 16S rRNA (E) sequencing. C) Rarefaction curves (minimum library size, total sum normalization). The relationship between the number of OTUs observed and the sequencing depth is shown for all samples.

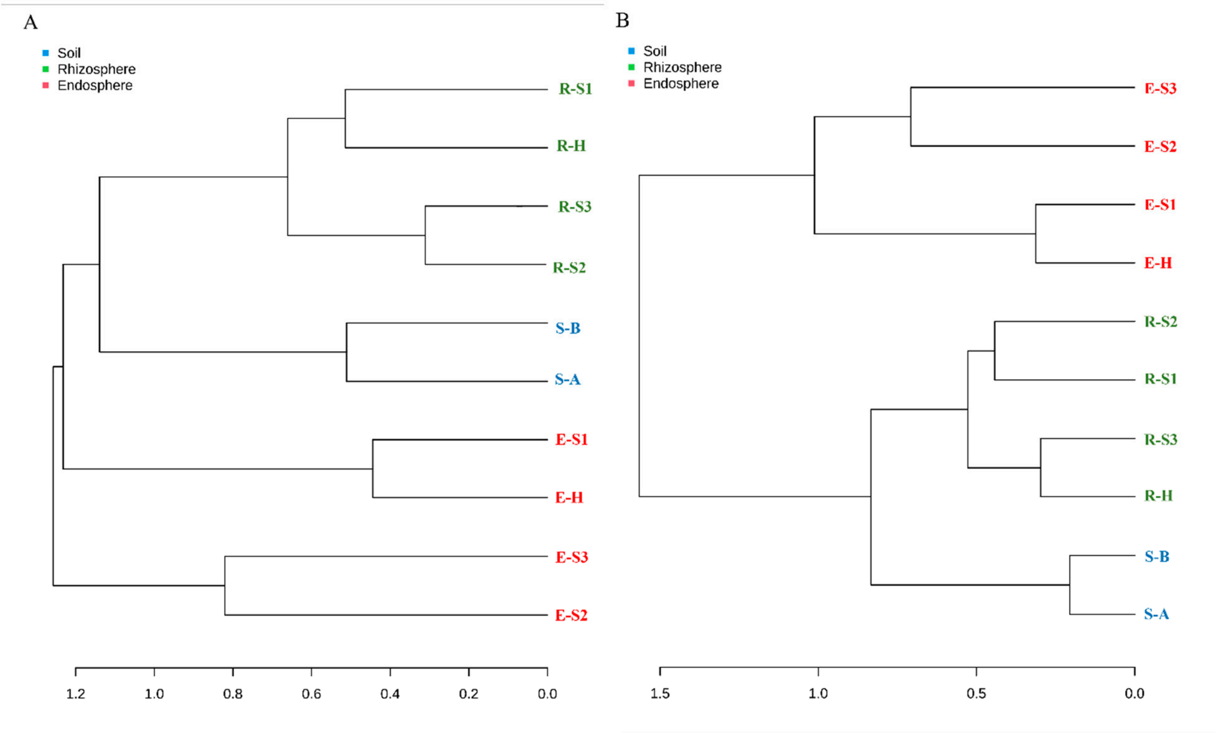

Supplemental Figure S2. Dendrogram analysis of biome data for mycobiome (A) and microbiome (B) using Bray-Curtis Index as a distance measure and Ward clustering algorithm.

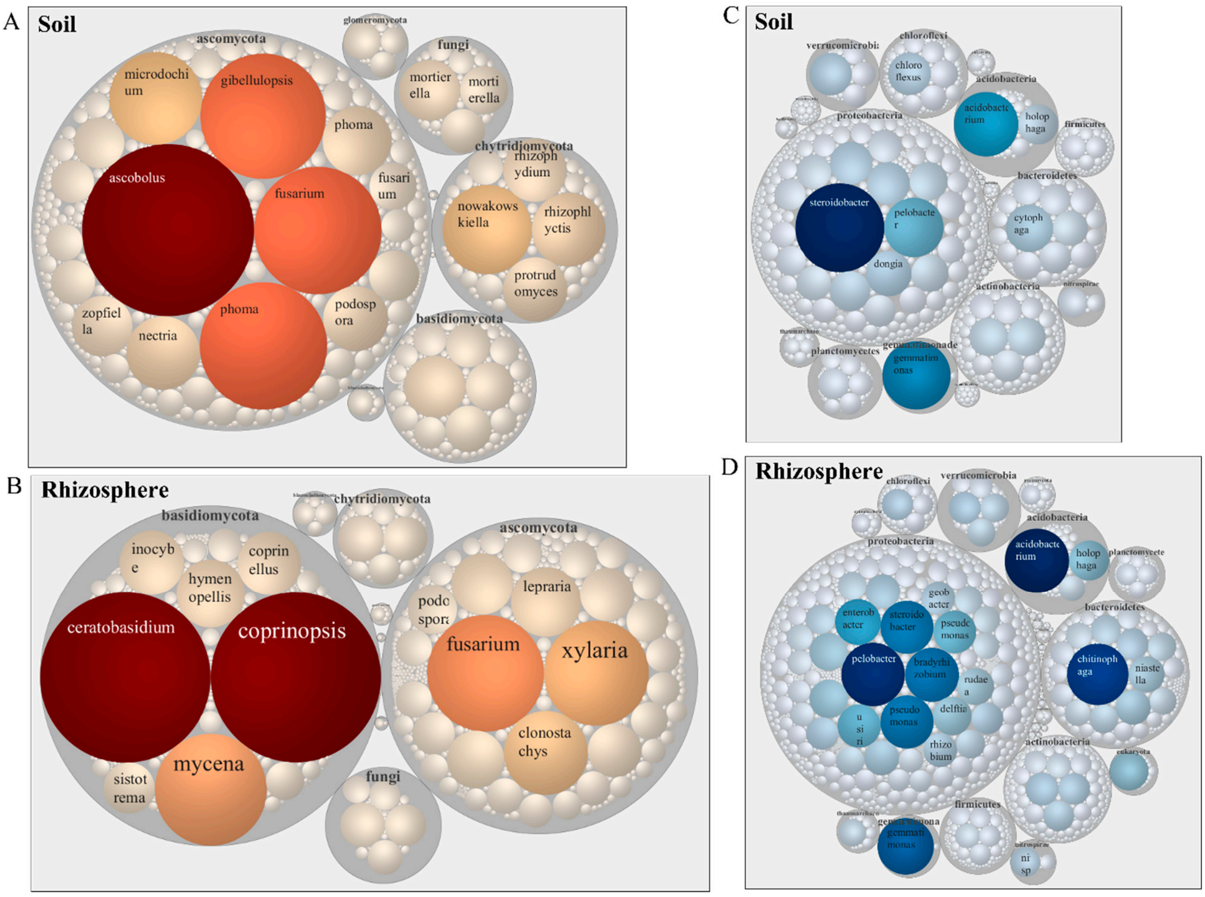

Supplemental Figure S3. A – D) Circular maps reporting taxonomic classification of fungal and bacterial taxa in bulk soil (A and C) and rhizosphere (C and D). Taxonomic differences are based on ITS and rRNA sequencing and OTUs with the highest abundance (n > 0.2 %) for fungal taxa (A and B) and bacterial taxa (C and D). The taxa are grouped by phylum; the labels show class, order, and species. The size of the map circle is proportional to the reads number.

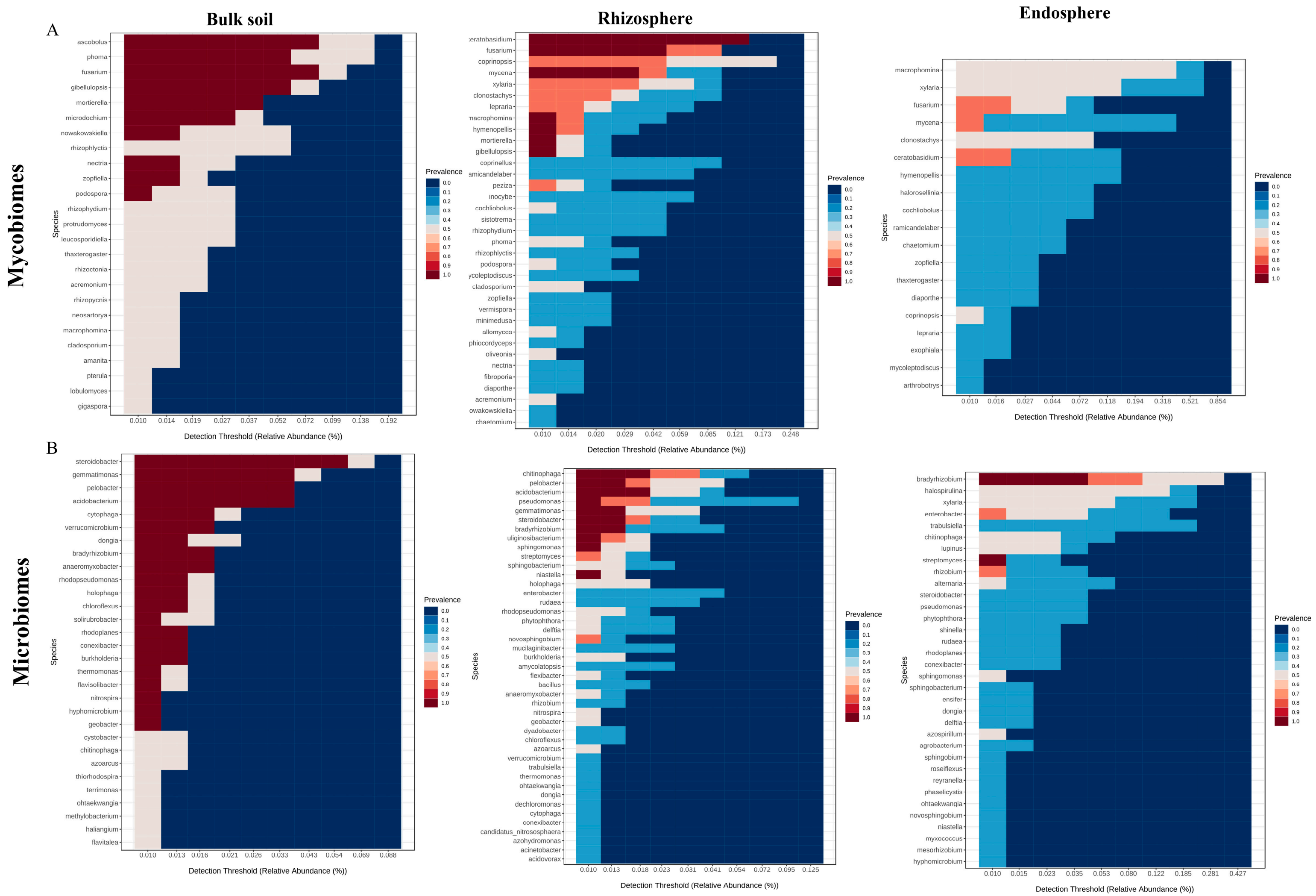

Supplemental Figure S4. Core biomes**.** Taxa detected containing classes with the highest prevalence (> 20%); count data were transformed to relative abundance for the analysis of the core mycobiome (A) in bulk soil, rhizosphere, and endosphere and the respective core bacterial microbiomes (B).

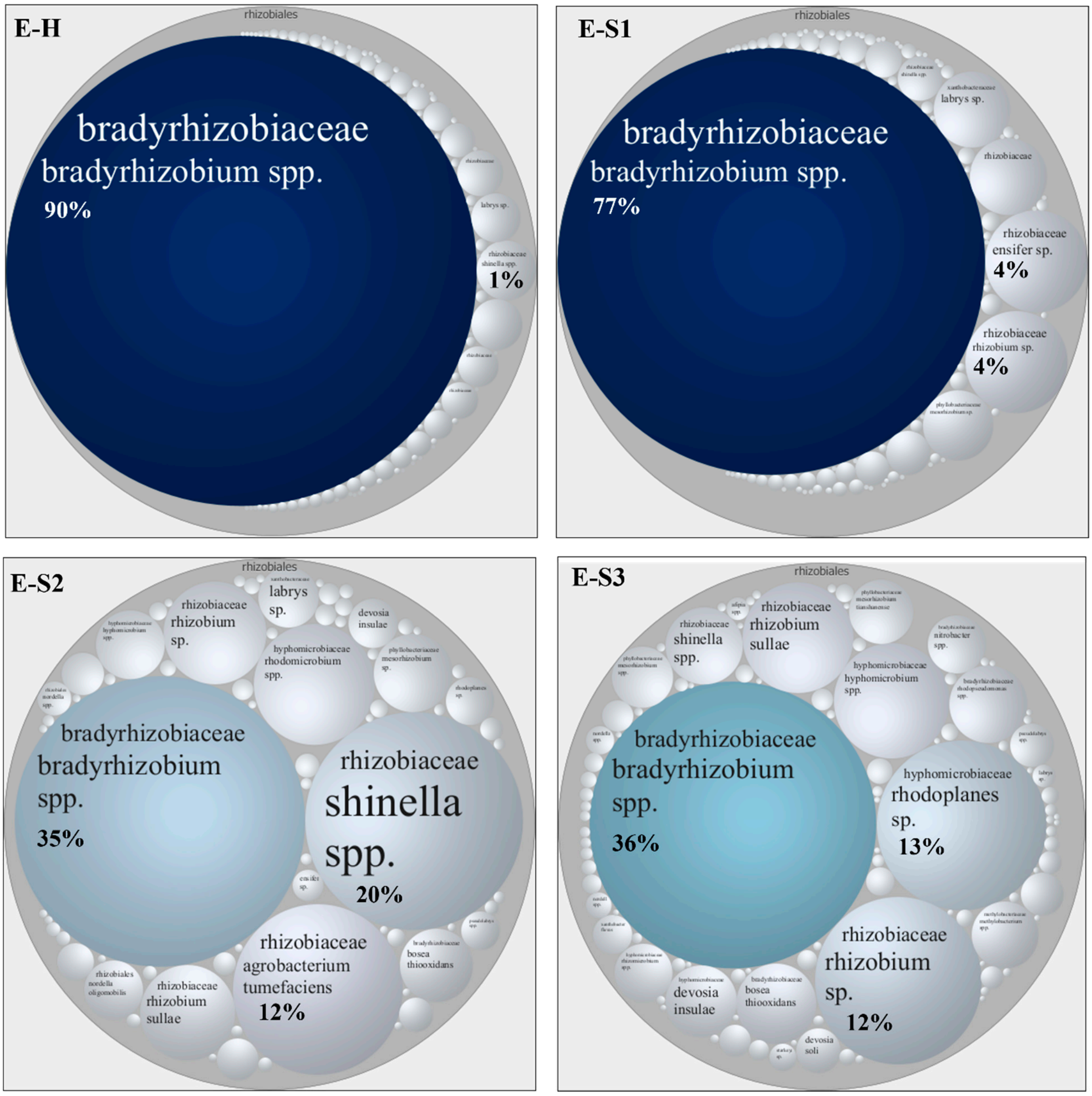

Supplemental Figure S5. Average reads numbers are based on 16S rRNA sequencing for the E-H (healthy) and TRD-symptomatic samples (E-S1, E-S2, and E-S3). Labels show family and species. Numbers depict percentages of selected taxonomical categories. The size of the map circle is proportional to the reads number.

A

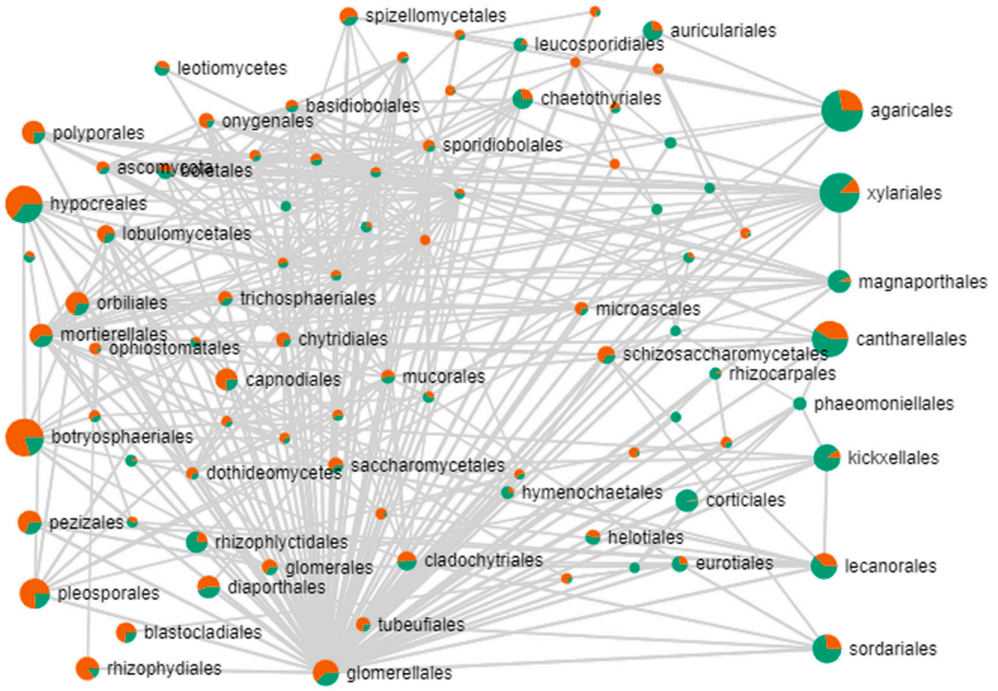
spizellomycetales
auriculariales
leucosporidiales
leotiomycetes
chaetothyriales
agaricales
basidiobolales
onygenales
polyporales
sporidiobolales
ascomycota
boletales
hypocreales
xylariales
lobulomycetales
magnaporthales
orbiliales
trichosphaeriales
microascales
mortierellales
ophiostomatales
chytridiales
cantharellales
schizosaccharomycetales
capnodiales
mucorales
rhizocarpales
phaeomoniellales
botryosphaeriales
kickxellales
dothideomycetes
saccharomycetales
hymenochaetales
corticiales
pezizales
rhizophlyctidales
helotiales
glomerales
cladochytriales
eurotiales
lecanorales
pleosporales
diaporthales
tubeufiales
blastocladiales
sordariales
rhizophydiales
glomerellales

B

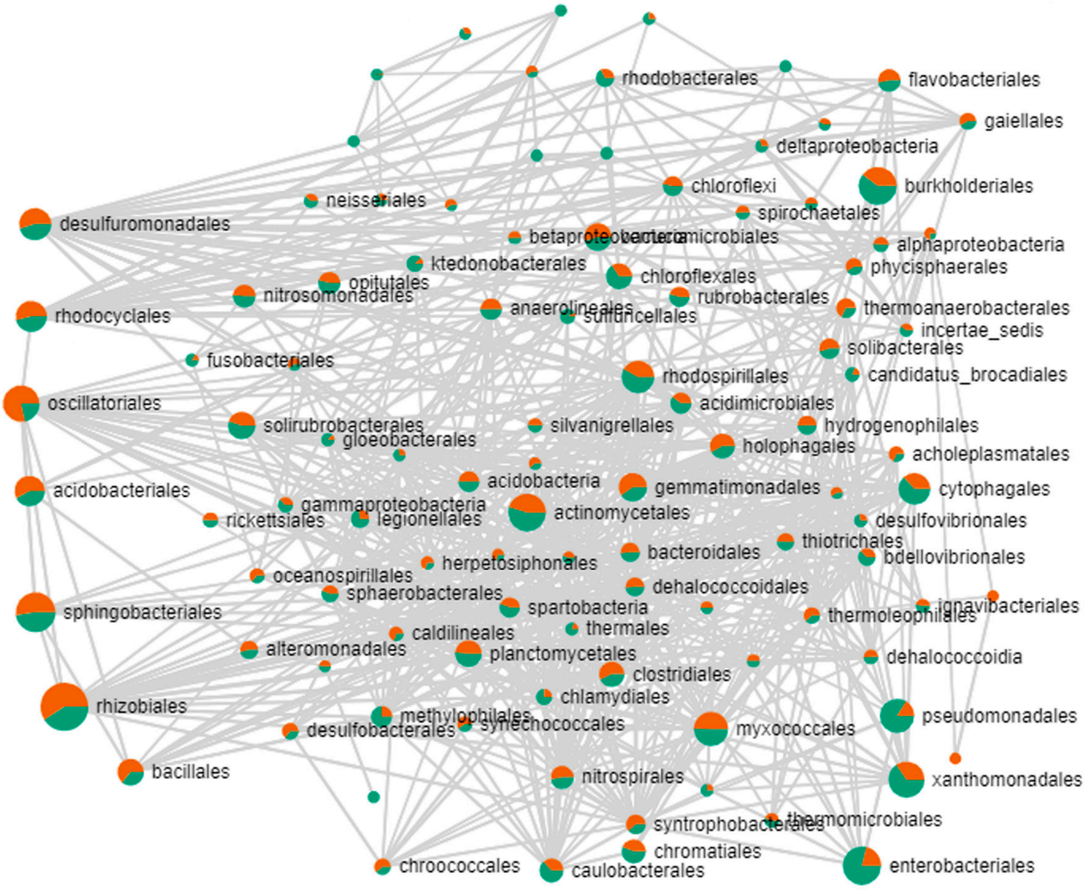
rhodobacterales
flavobacteriales
gaiellales
deltaproteobacteria
chloroflexi
burkholderiales
neisseriales
desulfuromonadales
spirochaetales
alphaproteobacteria
ktedonobacterales
phycisphaerales
chloroflexales
opitutales
nitrosomonadales
rubrobacterales
rhodocyclales
anaerolineales
sulfuricellales
thermoanaerobacterales
incertae_sedis
solibacterales
fusobacteriales
rhodospirillales
candidatus_brocadiales
oscillatoriales
acidimicrobiales
solirubrobacterales
silvanigrellales
hydrogenophilales
gloeobacterales
holophagales
acholeplasmatales
acidobacteria
gemmatimonadales
cytophagales
acidobacteriales
gammaproteobacteria
actinomycetales
rickettsiales
legionellales
desulfovibrionales
thiotrichales
bacteroidales
bdellovibrionales
herpetosiphonales
oceanospirillales
dehalococcoidales
sphaerobacterales
spartobacteria
sphingobacteriales
thermoleophilales
ignavibacteriales
thermales
caldilineales
alteromonadales
planctomycetales
dehalococcoidia
clostridiales
chlamydiales
rhizobiales
methylophilales
pseudomonadales
synechococcales
desulfobacterales
myxococcales
bacillales
nitrospirales
xanthomonadales
syntrophobacterales
thermomicrobiales
chromatiales
chroococcales
caulobacterales
enterobacteriales

Supplemental Figure S6. A correlation network was generated using the SparCC algorithm, with nodes representing taxa at the genus level and edges representing correlations between taxa pairs. Co-occurrence networks are based on the Pearson correlation of ITS (A) and 16S (B) rRNA reads extracted from metagenomes of healthy (purple) and TRD symptomatic (orange) plants and soil (green). A connection between nodes stands for a statistically significant ($p<0.05$) correlation with magnitude (correlation threshold) $r > 0.5$. Network parameters: p-value threshold 0.05, correlation threshold 0.3.
